# Health promotion for older adults in primary health care: practices and challenges

**DOI:** 10.15649/cuidarte.5409

**Published:** 2026-09-08

**Authors:** Fernanda Souza Tomé da Silva, Júlia Graciela de Souza, Luiz Eduardo Weimer, Regiane da Silva Macuch, Bianca Pereira de Assis, Aliny de Lima Santos

**Affiliations:** 1 Universidade Cesumar – UNICESUMAR, Itajaí, Brazil. E-mail: nandatome@gmail.com Universidade Cesumar Itajaí Brazil nandatome@gmail.com; 2 Universidade Cesumar – UNICESUMAR, Blumenau, Brazil. E-mail: nutrijuliagraciela@gmail.com Universidade Cesumar Blumenau Brazil nutrijuliagraciela@gmail.com; 3 Prefeitura de Navegantes, Navegantes, Brazil. E-mail: enf.luizweimer@gmail.com Prefeitura de Navegantes Navegantes Brazil enf.luizweimer@gmail.com; 4 Universidade Cesumar – UNICESUMAR, Maringá, Brazil. E-mail: regiane.macuch@unicesumar.edu.br Universidade Cesumar Maringá Brazil regiane.macuch@unicesumar.edu.br; 5 Universidade Cesumar – UNICESUMAR, Maringá, Brazil. E-mail: biiancaassis23@gmail.com Universidade Cesumar Maringá Brazil biiancaassis23@gmail.com; 6 Centro de Ciências da Saúde, Departamento de Enfermagem, Universidade Estadual de Maringá (UEM), Maringá, Brazil. E-mail: alsantos@uem.br Universidade Estadual de Maringá Maringá Brazil alsantos@uem.br

**Keywords:** Primary Health Care, Health Promotion, Public Health, Aged, Atención Primaria de Salud, Promoción de la Salud, Salud Pública, Anciano, Atenção Primária à Saúde, Promoção da Saúde, Saúde Pública, Pessoas Idosas

## Abstract

**Introduction:**

Health promotion actions in Primary Health Care (PHC) are essential for preventing health problems and improving the population’s quality of life, particularly among older adults. However, the implementation of these actions faces structural and organizational challenges.

**Objective:**

To map Health Promotion activities for older adults carried out in PHC in a municipality in Santa Catarina and to understand the challenges related to their implementation and execution from the perspective of practicing health professionals.

**Materials and Methods:**

This qualitative study was grounded in the constructivist paradigm and employed Fourth Generation Evaluation. Interviews were conducted with eight Family Health Strategy professionals, and a mapping of the collective activities offered was conducted. Data was collected between December 2024 and January 2025, and manually analyzed using the Constant Comparative Method, without the use of qualitative data analysis software.

**Results:**

The findings indicated difficulties in community engagement, limited resources, and weaknesses in the organization of health promotion actions for older adults. In addition, there was a predominance of initiatives focused on chronic diseases, while actions aimed at active aging remain underexplored.

**Discussion:**

The results highlight the need for continuous professional training, stronger coordination between management and health teams, and the adoption of more effective strategies to engage the community.

**Conclusion:**

Overcoming institutional and structural barriers is essential to ensure greater equity in access to preventive and health promotion practices.

## Resumen

**Introducción:**

Las acciones de promoción de la salud en Atención Primaria de Salud (APS) son fundamentales para prevenir problemas de salud y mejorar la calidad de vida de la población, especialmente de los adultos mayores. Sin embargo, la implementación de estas acciones enfrenta desafíos estructurales y organizativos.

**Objetivo:**

Mapear las acciones de promoción de la salud para adultos mayores realizadas en APS en un municipio de Santa Catarina y comprender los desafíos para su implementación y ejecución desde la perspectiva de los profesionales de la salud en activo.

**Materiales y métodos:**

Estudio cualitativo, basado en el paradigma constructivista y utilizando la Evaluación de Cuarta Generación. Se realizaron entrevistas a ocho profesionales de la Estrategia de Salud Familiar y se llevó a cabo una encuesta sobre las acciones colectivas desarrolladas. La recolección de datos se realizó entre diciembre de 2024 y enero de 2025, y los datos se analizaron mediante el Método Comparativo Constante.

**Resultados:**

Los resultados señalaron dificultades en la adherencia comunitaria, escasez de recursos y debilidades en la organización de las acciones de promoción de la salud dirigidas a adultos mayores. Además, predominaron las acciones enfocadas en enfermedades crónicas, mientras que las iniciativas dirigidas al envejecimiento activo están poco exploradas.

**Discusión:**

Se evidenció la necesidad de capacitación continua para los profesionales, mayor coordinación entre la gerencia y los equipos de salud, y la adopción de estrategias más efectivas de participación comunitaria.

**Conclusiones:**

Superar las barreras institucionales y estructurales es fundamental para garantizar una mayor equidad en el acceso a las prácticas preventivas y de promoción de la salud.

## Resumo

**Introdução:**

As ações de Promoção da Saúde na Atenção Primária à Saúde (APS) são fundamentais para prevenir agravos e melhorar a qualidade de vida da população, especialmente das pessoas idosas. No entanto, a implementação dessas ações enfrenta desafios estruturais e organizacionais.

**Objetivo:**

Mapear ações de Promoção da Saúde para pessoas idosas realizadas na APS de um município de Santa Catarina, e compreender os desafios para sua implementação e execução na perspectiva de profissionais de saúde atuantes.

**Materiais e métodos:**

Trata-se de um estudo qualitativo, fundamentado no paradigma construtivista e utilizando a Avaliação de Quarta Geração. Foram realizadas entrevistas com oito profissionais da Estratégia Saúde da Família e levantamento das ações coletivas desenvolvidas. A coleta de dados ocorreu entre dezembro de 2024 e janeiro de 2025, sendo os dados analisados manualmente pelo Método Comparativo Constante, sem utilização de software de análise qualitativa.

**Resultados:**

Os resultados apontaram dificuldades na adesão da comunidade, escassez de recursos, além de fragilidades na organização das ações de Promoção da Saúde voltadas às pessoas idosas. Além disso, observou-se a predominância de ações voltadas a doenças crônicas, enquanto iniciativas direcionadas ao envelhecimento ativo são pouco exploradas.

**Discussão:**

Observou-se a necessidade de capacitação contínua dos profissionais, maior articulação entre gestão e equipes de saúde e a adoção de estratégias mais efetivas de engajamento da comunidade.

**Conclusões:**

Concluiu-se que superar barreiras institucionais e estruturais é essencial para garantir maior equidade no acesso às práticas preventivas e promocionais.

## Introduction

Health Promotion (HP) is a fundamental strategy for improving the quality of life of populations, contributing to the reduction of inequalities and the prevention of health problems^1^. It also seeks to identify and address the factors that condition, facilitate, or hinder access to healthier lifestyle choices^2^.

Primary Health Care Units (PHCUs) serve as settings for the implementation of Primary Health Care (PHC) actions aimed at comprehensive care, the preservation of autonomy in healthy practices, and the reduction of health risks among the population^3^. When effectively implemented, these actions expand access to practices that promote physical, mental, and social well-being^3^.

Activities such as dance groups, physical exercise programs, group-based educational activities on healthy eating, and initiatives focused on medicinal plants and phytotherapy are examples of practices that have become established in Brazil as strategies that encourage the exchange of experiences while promoting quality of life and autonomy^4^. Older adults have particularly benefited from these initiatives, especially those designed to foster social interaction and maintain physical and mental health^4^.

Given the accelerated aging of the populationl^5^, discussions regarding the care of older adults within PHC have gained increasing relevance. Care is now understood beyond the mere absence of disease, with greater emphasis placed on the maintenance of functional capacity, making systematic and participatory PHC actions essential in this context^6^. Promoting autonomy, social participation, and the appropriate management of prevalent health conditions among older adults is therefore crucial to achieving these objectives^2^.

Despite these advances, the implementation of health promotion actions in PHC settings continues to face several barriers, including structural limitations, local work dynamics, and increasing population demand^7^. Difficulties in coordinating actions across different sectors of the health system negatively affect the continuity of care, contribute to service fragmentation, and hinder multisectoral collaboration with other areas of public administration, such as social assistance and sports^8^. Understanding these practices and identifying the factors that hinder their implementation provide opportunities to improve and redesign health promotion actions, strengthening existing strategies and fostering their development in contexts where they remain limited^9^.

Therefore, this study aimed to map HP actions for older adults developed within PHC in a municipality in the state of Santa Catarina, Brazil, and to understand the challenges related to their implementation and execution from the perspective of healthcare professionals involved in these activities.

## Materials and Methods

This is a qualitative, descriptive and evaluative study grounded in the constructivist paradigm and conducted in accordance with the Consolidated Criteria for Reporting Qualitative Research (COREQ). As its theoretical and methodological framework, the study adopted Fourth Generation Evaluation (FGE), proposed by Guba and Lincoln^10^, which is characterized as a participatory, dialogical and responsive approach. FGE guides the entire investigative process, from the formulation of research questions to the analysis, interpretation and validation of findings, through continuous negotiation between researchers and participants. It values multiple perspectives and recognizes context as a determinant of experience, thereby guiding the analysis of the claims, concerns, and issues (CCIs) of the groups involved^10^.

This study is part of a doctoral research project entitled “Challenges and Opportunities for Health Promotion Among Older Adults in Primary Health Care: A Fourth-Generation Evaluation.” The research was conducted in the Primary Health Care (PHC) network of a municipality located on the northern coast of Santa Catarina, Brazil. The municipality was selected due to its accelerated population growth and significant aging process, characteristics that make it a relevant setting for analyzing health promotion actions aimed at older adults within PHC.

According to data from the 2022 Demographic Census, the municipality had 86,401 residents, including 11,166 older adults, corresponding to approximately 13% of the total population. For 2025, the population is estimated to exceed 96,000 inhabitants, reinforcing the demographic and epidemiological transition observed in the region^11^. PHC coverage is universal through the Family Health Strategy (FHS), comprising 18 Primary Health Care Units (PHCUs) distributed across 28 Family Health Teams (FHTs). This setting therefore provides a favorable context for investigating the organization of health promotion actions and the challenges associated with promoting active and healthy aging.

Data collection was organized into two main stages:

1. Initial Mapping: In December 2024, in-person visits were conducted to all 18 Primary Health Care Units (PHCUs) in the municipality to identify the health promotion actions implemented across different life stages, particularly those aimed at older adults. Semi-structured interviews were conducted with managers or designated healthcare professionals to gather information on the actions performed, duration of implementation, average number of participants, professionals involved, and resources used. Participants were also asked to provide objective descriptions of the activities and their target populations. This stage preceded the Hermeneutic-Dialectic Circle (HDC).

Following this phase, eight PHCUs were identified as not offering specific collective actions targeted at older adults. One of these units was randomly selected for further investigation during the subsequent qualitative stage. The mapping process generated a descriptive overview of the actions developed within the municipality and supported the selection of the PHCU included in Stage 2.

2. Hermeneutic-Dialectic Circle (HDC): The selected PHCU operates under the Family Health Strategy (FHS) model and comprises two Family Health Teams (FHTs). It is located in an area characterized by high social vulnerability, sociocultural diversity, and a substantial older adult population. Because the unit did not offer specific collective health promotion actions for older adults, it provided an opportunity to gain a deeper understanding of the challenges, limitations and perceptions of healthcare professionals regarding the implementation of collective health promotion initiatives aimed at active and healthy aging within PHC.

The HDC began with an interview with the unit manager, who formally received the invitation to participate and subsequently shared it with the healthcare team. Individual semi-structured interviews were then conducted sequentially, with each participant identifying the next interviewee. This procedure enabled the progressive incorporation of new questions emerging from previously conducted interviews^10^.

Eligibility criteria included healthcare professionals who had worked at the selected unit for at least six months. Professionals who were on vacation, leave of absence, or medical leave during the data collection period were excluded.

The interviews were conducted individually, beginning with the guiding question: “What are the main challenges you identify in this service regarding Health Promotion with a focus on healthy aging?”. As the Hermeneutic-Dialectic Circle (HDC) progressed, additional probing questions were incorporated, resulting in a total of ten questions by the end of the process. Interviews were conducted at the PHCU facilities, at previously scheduled times, between December 2024 and January 2025. Data collection was concluded when the constructions became stable and no new information relevant to the study objectives emerged^10^.

Eight healthcare professionals representing different categories of the multidisciplinary team participated in the HDC, including nurses, physicians, community health workers, nursing technicians and a receptionist. The participants had an average length of service of 50 months.

The interviews were audio-recorded with participants' consent and transcribed verbatim using Microsoft Office 365. Only minor linguistic adjustments were made to improve readability while preserving the original meaning of the statements. The interviews lasted approximately 30 minutes on average. Following transcription, the transcripts were returned to participants for review and validation, ensuring the accuracy and credibility of the information collected.

Data analysis followed the Constant Comparative Method^10^, pwhich is specific to Fourth Generation Evaluation. The transcripts were manually coded line by line, without the use of qualitative data analysis software, and continuously compared throughout the analytical process. Units of meaning were grouped and reorganized through an iterative and dialogical process into provisional constructions based on their similarity, frequency and relevance to the study objectives.

To complement the interviews, participant observation was conducted for a total of 80 hours at the selected PHCU. This stage enabled a more comprehensive understanding of service dynamics and of the challenges involved in promoting the health of older adults within the PHC context.

Subsequently, a negotiation session was conducted with the eight professionals interviewed. Held in March 2025 and lasting approximately two hours, the session aimed to present, discuss and validate the provisional constructions, as well as to reach consensus regarding the interpretations and findings generated throughout the study.

In addition, the Ishikawa diagram^12^ was used as an analytical tool to visually synthesize the challenges identified in the implementation of Health Promotion (HP) actions aimed at active and healthy aging. Constructed from data collected at the PHCU selected for the Hermeneutic-Dialectic Circle (HDC) and refined during the negotiation session, the diagram enabled the systematic organization of factors associated with the difficulties in implementing these actions within the local context, according to the collective perceptions of the participating professionals.

To enhance textual clarity, grammar, cohesion, and the preparation of the English and Spanish abstracts, ChatGPT-4 was used as an editorial support tool. This resource was employed exclusively to assist in manuscript editing and language refinement and did not interfere with data analysis, interpretation, or the development of the study findings^13^.

This study was approved by the Research Ethics Committee of the Central Education Unit of Faem Faculty (UCEFF) under CAAE No. 84997124.3.0000.8146. In addition, authorization to conduct the study was granted by the Municipal Health Department for implementation within the municipality's Primary Health Care Units (PHCUs). All participants received and signed an Informed Consent Form (ICF), ensuring confidentiality, anonymity and voluntary participation in accordance with current ethical guidelines. Participant anonymity was preserved using pseudonyms derived from Greek mythology. All data generated and analyzed during the study are publicly available through Mendeley Data^14^.

## Results

The analysis revealed several challenges related to health promotion for older adults within Primary Health Care (PHC). Among the 18 Primary Health Care Units (PHCUs) in the municipality, four did not carry out any collective health promotion actions for any population group during the period analyzed, providing only individual consultations and routine care. These units operated exclusively under the Primary Care Team (PCT) model.

The remaining units developed collective actions targeting different population groups, including pregnant women, individuals receiving mental health care, smoking cessation participants, and activities linked to the School Health Program (PSE), but without specific initiatives aimed at older adults. Only ten PHCUs implemented health education actions targeting older adults, such as walking groups, community gardening initiatives, and educational meetings. Among these, five offered health education groups for individuals with hypertension and diabetes mellitus. Although organized around the management of chronic conditions, these groups were included in the study because their participants were predominantly older adults Table 1.

The actions varied in terms of frequency, number of participants, and professional involvement, with nurses and community health workers being the professionals most frequently engaged in these initiatives Table 1. Resource availability also differed among the units, ranging from materials supplied by the Municipal Health Department to the use of locally available resources and alternative community spaces Table 1.

**Table 1:** Mapping of Health Promotion actions for older adults in Primary Health Care Units in a municipality in Santa Catarina, Brazil. Navegantes, Santa Catarina, 2025

PHCU	Team Composition	Health Promotion Actions	Frequency	Mean Number of Participants	Professionals Involved	Resources Used
PHCU 2	3 FHT 1 PCT	Health education group for individuals with arterial hypertension and diabetes mellitus.	Monthly	5	Nurse	Materials provided by the Municipal Health Department (SVF foam sheets, cardboard, A4 paper)
PHCU 3	2 FHT	Health education group for individuals with arterial hypertension and diabetes mellitus.	Monthly	16	Nursing technician, community health worker and dentist	Refreshments and gifts (self-funded); office supplies and projector provided by the Municipal Health Department
PHCU 4	1 FHT	Health education group for individuals with arterial hypertension and diabetes mellitus.	Bimonthly	15	Community health worker, physician, dentist and nursing technician	Refreshments and gifts (self-funded); office supplies, WhatsApp and projector provided by the Municipal Health Department
PHCU 5	2 FHT	Walking group	Weekly	3	Community health worker, physician, nutritionist, dentis and nursing technician	Meeting room, gifts, seedlings, and refreshments (self-funded); office supplies and projector provided by the Municipal Health Department
PHCU 6	2 FHT	Walking group	Semiannual	5	Community health worker	Computer and sphygmomanometers
PHCU 12	1 FHT	Community garden	Monthly	2	Nursing technician	Seedlings (self-funded)
PHCU 15	2 FHT	Walking group	Weekly	10	Community health worker	CRAS facilities; materials provided by the Municipal Health Department (cardboard, SVF foam sheets and A4 paper)
PHCU 16	3 FHT	Walking group	Weekly	10	Nurses and community health workers	Scale, measuring tape, projector and microphone
PHCU 17	1 FHT	Health education group for individuals with arterial hypertension and diabetes mellitus.	Annual	4	Nurses, dentist, physician and nursing technician	Projector, materials provided by the Municipal Health Department, measuring tape and scale
PHCU 18	3 FHT	Walking group; health education group for individuals with arterial hypertension and diabetes mellitus; community garden.	Weekly (walking group); biweekly (health education group); community garden under implementation	15 (walking group); 10 (health education group); community garden under implementation	Nurses, dentist, community health worker and nursing technician	Glucometer, sphygmomanometers and materials provided by the Municipal Health Department (cardboard, SVF foam sheets and A4 paper)

Note: PHCU: Primary Health Care Units; FHT: Family Health Team; PCT: Primary Care Team; CRAS: the Brazilian Social Assistance Reference Center; SVF: Satin Vinyl Foam; A4 paper: ISO 216 A4 paper format (210 × 297 mm).

The mapping of Health Promotion (HP) actions targeting older adults identified their distribution, frequency, professionals involved, and resources mobilized across the Primary Health Care Units (PHCUs). Subsequently, a qualitative analysis of the accounts provided by professionals from the selected unit was undertaken to further explore the challenges related to the implementation of HP actions in the Primary Health Care setting Table 2.

**Table 2:** Characteristics of the professionals participating in the Hermeneutic-Dialectic Circle at a Primary Health Care Unit. Navegantes, Santa Catarina, Brazil, 2025

Codename	Professional Category	Age (years)	Sex	Length of Experience in PHC (months)	Length of Service in the Territory (months)
Aphrodite	Nursing technician	51	Female	73	36
Apollo	Community health worker	46	Female	9	9
Artemis	Nurse	39	Male	156	36
Athena	Nurse	29	Female	7	7
Ivy	Physician	34	Female	48	48
Minotaur	Community health worker	52	Female	9	9
Persephone	Receptionist	47	Female	6	6
Zeus	Community health worker	54	Female	252	252

Note: PHC: Primary Health Care

These challenges emerged in the form of claims, concerns, and issues (CCIs), which were grouped into provisional constructions and subsequently validated during the negotiation session. The challenges were organized into four main constructions—team, community, resources and management—and systematized using an Ishikawa diagram^12^, as presented in Figure 1.

**Figure 1:**
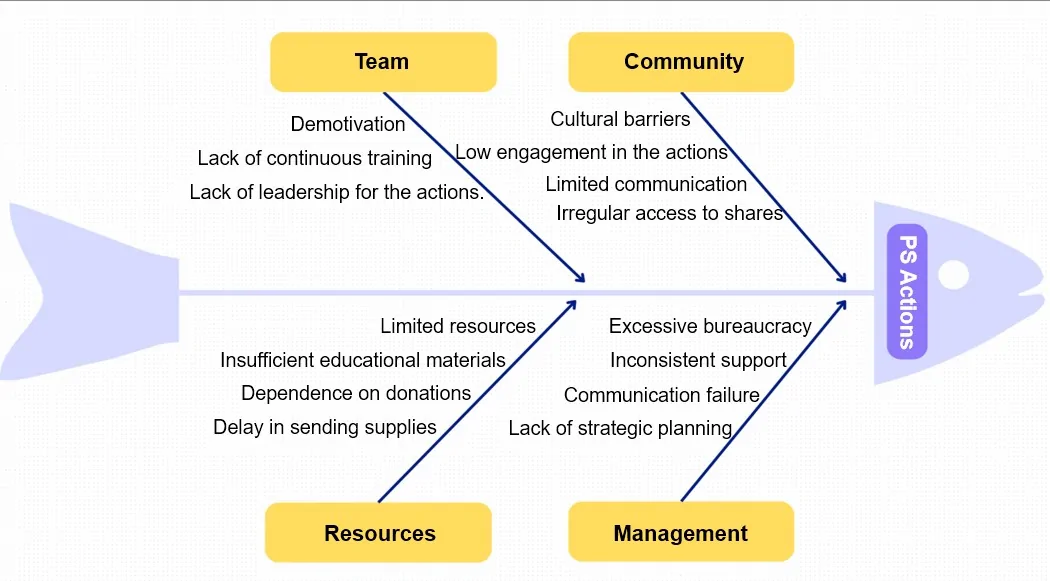
Ishikawa diagram

*Note: Illustrating the main challenges associated with the implementation of Health Promotion actions for older adults in Primary Health Care, organized into four categories: team, community, resources, and management. Navegantes, Santa Catarina, Brazil, 2025.*

To illustrate the four main constructions identified through the analysis and systematized in the Ishikawa diagram, illustrative excerpts from the participants' statements are presented below.

***“I have the desire, but I would also need the knowledge”*: Challenges in healthcare teams’ planning and implementation of Health Promotion actions.**

The actions developed by the healthcare teams were predominantly focused on preventing complications, particularly those associated with the most prevalent chronic conditions among older adults. The incorporation of broader Health Promotion approaches remained limited, restricting the scope and potential impact of these practices within the routine activities of the services.

*“The actions we carry out are intended to prevent further harm to patients’ health. [...] In this way, we avoid having more cases of chronic conditions such as hypertension, diabetes, and obesity [...] and help keep the population healthier [...]” (Hera)*

Furthermore, the participants’ reports highlighted operational difficulties that affect the work of healthcare teams. Staff shortages and professional demotivation limit the planning, implementation, and continuity of collective Health Promotion actions.

*“[...] there are professionals who are demotivated [...] there is a lack of incentive, we need incentive [...]”.(Apollo)*

*“[...] there are few of us and so sometimes we fail to do certain things due to lack of time [...]”. (Aphrodite) *

*“[...]this is hampered by the demand for acute problems, which we end up having to solve [...].” (Hera)*

Nevertheless, leadership emerged as a fundamental element in the organization and motivation of healthcare teams. Participants highlighted the absence of technical leadership and qualified support to guide the planning and implementation of Health Promotion actions, particularly those aimed at promoting active and healthy aging.

*“[...] what’s missing is someone who [...] has leadership skills [...] who will pull the team along and make things happen. Because the team is very cohesive, but without someone to organize, the actions don’t hold up.” (Minotaur)*

*“[...] We will perhaps need people who already have experience to pass it on to us [...] We want to do things differently, but often we don't know where to start. [...] we even try, but without this support it's difficult to continue.” (Apollo)*

*“[...] The biggest challenge is this, [to] make [...] it happens.” (Minotaur)*

**“Trying to convince patients to adhere”: Low adherence as a barrier imposed by the community**

Low participation among older adults emerged as one of the main challenges faced by healthcare teams. This recurring issue compromises both the continuity and effectiveness of Health Promotion actions. In many cases, the challenge manifests as difficulties in engaging and mobilizing the community to participate in the proposed activities.

*“[...] the most difficult thing is trying to convince patients to adhere [...]”. (Minotaur)*

*“[...] There’s no way we can carry out Health Promotion actions if we don’t have community support [...]”.(Hera)*

*“[…] currently, participation is low […] not [...] because of the users […] but because of the few activities they are carrying out at this moment […]”. (Hera)*

Professionals also attributed low participation to weak bonds between older adults and the health unit. The lack of collective spaces for interaction and the difficulty in establishing lasting relationships were identified as factors that limit engagement over time. Furthermore, socioeconomic conditions were reported as additional barriers affecting access to services and participation in Health Promotion activities.

*“[...] there’s that issue of the groups [...] when we manage to rescue them [...] it will be more effective [...] to create stronger bonds [...]”. (Athena)*

*“[...] the difficulty of engagement [...] on the part of the community [...]”. (Artemis)*

*“[...] our community is needy [...] sometimes it doesn't even have money to pay for the bus [...]”. (Zeus)*

**“We have to pay for everything out of our own pockets”: Resource constraints as a barrier to Health Promotion actions**

The scarcity of supplies, materials, and structural support was identified as a recurring obstacle to the implementation of Health Promotion actions, directly affecting the work of healthcare teams.

*“[...]here, sometimes, we don’t have many resources[...]”. (Aphrodite)*

*“[...] difficulty [...] of materials and equipment for some activities that we might want to develop [...]”.(Ártemis)*

In addition to operational limitations, participants highlighted the lack of consistent institutional and structural support. In some cases, this shortcoming required healthcare professionals to cover basic expenses using their own resources to enable the implementation of Health Promotion actions, contributing to professional exhaustion and feelings of institutional neglect.

*“[...] what we lack is the collaboration of management for materials [...] offering coffee, souvenirs [...] because we have to pay for everything out of our own pockets [...]”. (Zeus)*

Within the daily routine of the service, insufficient structural support permeates work processes and directly affects the implementation of Health Promotion actions. Limited access to basic materials shifts responsibility for enabling these initiatives to healthcare teams, even though such actions should be supported institutionally.

*“[...] if I need to print material [...] it will all be black and white [...] a folder like that doesn't attract attention [...] we would need support [...] of material [...]”. (Apollo)*

*“[...] I think that improving things would be having free access to materials [...] scheduling appointments and not lacking anything [...] because sometimes there is a lack of material [...]”. (Zeus)*

**“We need to be heard first”: Communication barriers and the bureaucratization of management**

Within the management context, participants’ accounts revealed that weak institutional communication and bureaucratic decision-making processes constitute important barriers to the implementation of Health Promotion actions. Management was perceived as being distant from the daily realities of healthcare teams, particularly regarding actions aimed at promoting active and healthy aging among older adults.

*“[...] sometimes what doesn't depend on our primary health care unit, what depends on outside, on the management of the Secretariat, takes time” (Zeus)*

*“[...] there’s no way we can carry out Health Promotion actions if we don’t have support from the city hall [...]”. (Hera)*

Participants’ accounts also revealed the absence of systematic opportunities for dialogue and meaningful engagement with management. This limitation reduces team autonomy and hinders the implementation of actions within the territory, particularly those that require planning, coordination, and continuity.

*“I want to form my group [...], but I need my superior to say: 'go ahead, form your group' [...] so we need that, we need to be heard first [...]”. (Apollo)*

*“[...] there's a lack of feedback [...] of knowing if what we ask for will be granted [...]”. (Athena)*

*“[...] we speak, but many times it seems like it doesn't get us anywhere [...]”. (Apollo)*

## Discussion

The Health Promotion actions implemented in Primary Health Care Units (PHCUs) revealed considerable heterogeneity in the activities offered, reflecting conceptual and policy-related challenges in defining and operationalizing Health Promotion^6^. A predominance of practices focused on specific conditions, particularly Noncommunicable Chronic Diseases (NCDs), was identified. These practices remain largely anchored in a biomedical model that prioritizes the complaint–conduct approach. Such a perspective limits the incorporation of actions addressing the social determinants of health and maintains Health Promotion as primarily associated with the prevention of health problems, which is inconsistent with the principles of the National Health Promotion Policy (NHPP)^15^.

This pattern has also been described in other studies, which report the widespread implementation of actions linked to the School Health Program (SHP), groups for pregnant women and health education groups for individuals with hypertension and diabetes within Primary Health Care (PHC)^16^,^17^. In the present study, this configuration reinforces the relative invisibility of older adults, as the few initiatives identified as promoting active and healthy aging were limited to incipient actions such as walking groups, community gardens, and health education activities. Although relevant, these practices still lack strategic planning, institutional support, and a broader conceptual foundation, thereby limiting their potential to promote autonomy, functional capacity, and quality of life in later life^18^,^19^.

Although the Brazilian Unified Health System (SUS) has a specific policy to guide Health Promotion actions^6^, these practices remain largely focused on the prevention of complications and the control of noncommunicable chronic diseases (NCDs) in the daily routine of health services^20^. In the present study, actions targeting older adults were predominantly directed toward the management of chronic conditions, with limited incorporation of strategies aimed at promoting autonomy, functional capacity, and active aging^3^.

Working conditions emerged as a structuring factor in this context. Professional demotivation and the lack of institutional support hinder the organization, implementation, and continuity of collective actions, particularly those targeting older adults^21^. These challenges are compounded by weaknesses in professional training, as participants reported feeling insufficiently prepared despite their willingness to adopt different approaches. This finding reinforces the need for greater technical support, continuing education opportunities, and spaces for professional exchange and critical reflection. As a result, many actions remain confined to traditional formats focused on the transmission of information, with limited opportunities for dialogue and the co-construction of knowledge and practices with the community^22^.

National guidelines emphasize that Health Promotion actions should be developed with the community rather than for the community^4^, erequiring the overcoming of top-down approaches that are insensitive to the experiences, needs, and realities of the territory. Furthermore, community participation, particularly among older adults, contributes to empowerment, self-care, and improvements in quality-of-life^23^.

In this context, leadership emerged as a critical issue. Although participants reported positive teamwork and collaborative relationships, they also expressed the need for leaders capable of driving, coordinating, and sustaining initiatives over time. In the absence of such leadership, actions become excessively dependent on individual motivation and are therefore more vulnerable to the challenges and fluctuations of daily practice^9^.

Regarding the community, low participation in Health Promotion activities emerged as one of the main challenges faced by healthcare teams. Reduced participation among older adults has already been identified in the literature as an obstacle to the continuity and effectiveness of such activities, highlighting limitations in the ways these actions are integrated into and appropriated by the community^24^. These findings reinforce that participation should not be understood solely as an individual decision, but rather as an expression of broader social, cultural and organizational conditions. Although participants did not explicitly use the term “cultural barrier,” their accounts revealed difficulties related to how Health Promotion actions interact—or fail to interact—with the lifestyles, expectations, values, and reference frameworks of the community.

Previous studies indicate that low participation is also associated with concrete everyday factors, such as geographic barriers, transportation difficulties, and limited access to services, particularly in contexts of greater social vulnerability^5^-^7^. Furthermore, participation tends to be lower when individuals do not perceive clear benefits from the activities offered or have limited confidence in their effectiveness. These aspects are closely associated with levels of satisfaction and the bonds established between users and healthcare teams^1^-^4^.

The methodology adopted in Health Promotion actions is also known to directly influence this scenario. In the context of the present study, isolated and traditional practices demonstrated limited potential for community engagement, as they tend to reinforce vertical relationships and the passive role of users in the educational process^23^. In contrast, interventions oriented toward autonomy, empowerment, and active participation promoted greater engagement, fostering dialogue between scientific and popular knowledge while strengthening the bonds between healthcare teams and the communities they serve^25^.

For older adults, this perspective is particularly relevant. By recognizing individual trajectories, lived experiences, and social contexts, such approaches contribute to individuals perceiving themselves as active participants in their own care. This perspective is consistent with the principles of Health Promotion, which recognize social participation as a fundamental component of health-promoting practices^25^.

Resources—whether material, financial, or structural—are fundamental for the implementation of Health Promotion (HP) actions within Primary Health Care (PHC)^26^. However, participants’ accounts highlighted that the scarcity of these resources constitutes a recurring barrier, compromising both the continuity and quality of the initiatives developed. Resource limitations not only affect the implementation of actions but also restrict opportunities for innovation and the adaptation of initiatives to local needs^27^.

Insufficient resources also influence professionals’ perceptions of the value and feasibility of their work. The lack of adequate materials and infrastructure tends to generate feelings of devaluation and frustration, negatively affecting team motivation and the quality of the practices developed desenvolvidas^15^-^28^. This scenario highlights the need for stronger coordination between management and healthcare teams to identify gaps and develop solutions that are responsive to local realities^15^.

To address these challenges, adequate planning is essential, with priority given to strengthening infrastructure and providing support for healthcare teams. Furthermore, the implementation of participatory strategies may broaden the impact of Health Promotion (HP) initiatives and enhance their sustainability^23^.

Regarding older adults, the lack of managerial support and organized strategies has directly affected the continuity of actions aimed at promoting active and healthy aging. The absence of institutional responsiveness and support has hindered initiatives that could foster social participation, autonomy, and functional capacity in later life—fundamental pillars of healthy aging and quality of life. In this sense, Health Promotion actions targeting older adults require strategic planning and institutional coordination that consider their specific needs and recognize the territory as a space for the collective construction of health.

Management plays a central role in defining priorities, allocating resources, and sustaining actions developed within Primary Health Care (PHC)^19^. When active aging is not established as a strategic priority within institutional agendas, Health Promotion actions targeting older adults tend to assume a sporadic and fragmented character. In such situations, these initiatives become excessively dependent on the individual efforts of healthcare professionals^15^, compromising their continuity and reach. This scenario weakens PHC practices and reinforces the invisibility of older adults in the planning and implementation of collective actions^17^.

The bureaucratization of decision-making processes and delays in institutional responses discourage Health Promotion initiatives within Primary Health Care, particularly those that require intersectoral coordination and sustained support^24^. Strengthening participatory management through greater interaction between decision-making levels and local teams may foster more consistent actions that are better aligned with the needs of healthy aging within PHC^25^.

Although this study allowed for an in-depth analysis of Health Promotion practices within Primary Health Care, some limitations should be acknowledged. The sample consisted of healthcare professionals from a single municipality and a single Primary Health Care Unit (PHCU), which may limit the transferability of the findings to other contexts. Furthermore, data collection was restricted to healthcare professionals and did not include the perspectives of service users. Future studies should incorporate the views of older adults and other community members, as well as explore different regional contexts, to provide a more comprehensive understanding of the challenges and opportunities associated with Health Promotion in Primary Health Care.

## Conclusions

The study findings demonstrated that, although initiatives aimed at promoting active and healthy aging exist within Primary Health Care (PHC), they remain sporadic, poorly systematized, and frequently constrained by structural, organizational, and managerial barriers. A biomedical model continues to predominate, with practices focused primarily on chronic conditions, while actions aimed at promoting functional capacity, quality of life, autonomy, and empowerment among older adults remain limited.

The findings highlight the need to strengthen the role of older adults in their own care through improved institutional coordination and greater responsiveness and dialogue between healthcare teams and management. Such efforts may facilitate the planning and implementation of Health Promotion actions, create conditions for greater empowerment and active participation among older adults, and consolidate practices that value autonomy, self-care and community participation.
